# Comparison of Brucellosis and Rickettsiosis in Children: A Retrospective Cohort Study

**DOI:** 10.3390/jcm14051465

**Published:** 2025-02-21

**Authors:** Idan Lendner, Moshe Shmueli, Siham Elamour, Galina Ling, Shalom Ben-Shimol

**Affiliations:** 1 Faculty of Health Sciences, Ben-Gurion University of the Negev, Beer Sheva 84101, Israel; idanlend@post.bgu.ac.il (I.L.); mosheshm@post.bgu.ac.il (M.S.); sihame@clalit.org.il (S.E.); galinali@clalit.org.il (G.L.); 2 Pediatric Infectious Disease Unit, Soroka University Medical Center, Beer Sheva 84101, Israel; 3 The Pediatric Day-Care Unit, Soroka University Medical Center, Beer-Sheva 84101, Israel

**Keywords:** rickettsiosis, brucellosis, zoonosis, endemic diseases, febrile illness

## Abstract

**Background:** Fever of Unknown Origin (FUO) is a diagnostic challenge in pediatrics, often stemming from zoonotic infections. In southern Israel, brucellosis and rickettsiosis are endemic and share overlapping clinical features, making diagnosis difficult. We compared the demographic, clinical, and laboratory characteristics of pediatric brucellosis and rickettsiosis to aid in distinguishing between these diseases and guide early empirical treatment. **Methods:** We performed a retrospective cohort study, conducted between 2005 and 2020, on children who tested positive for either rickettsia or brucella. Data on demographic, clinical, laboratory, treatment, and outcome parameters were analyzed using descriptive, univariate, and multivariate statistical methods. **Results:** Overall, 775 patients were included, 440 with brucellosis and 335 with rickettsiosis. The majority of patients were of Bedouin ethnicity (99.5% and 90.7%, respectively). In univariate analysis, brucellosis was associated with male gender, young age, limping, anemia, and prolonged hospitalization duration. Rickettsiosis was characterized by high-grade fever, rash, headache, thrombocytopenia, elevated C-reactive protein (CRP), and gastrointestinal, respiratory, and neurological symptoms. Mortality rates were low in both groups (≤0.5%). In multivariate analysis, brucellosis was associated with limping (odds ratio = 7.27; with 95% confidence interval of 5.15–10.38), hemoglobin <10 mg/dL (2.01; 1.14–3.64), age <5 years (1.95; 1.25–3.07), warm season (1.84; 1.31–2.59), and male gender (1.57; 1.10–2.25). Rickettsiosis was associated with a rash (9.06; 3.91–24.9), CRP ≥5 mg/dL (4.03; 1.86–9.81), headache (3.01; 1.75–5.30), thrombocytopenia (2.61; 1.23–6.06), leukopenia (1.88; 1.19–2.98), and temperature ≥39.0 °C (1.66; 1.03–2.68). **Conclusions:** Brucellosis and rickettsiosis differ demographically and clinically in FUO cases. These findings highlight the importance of distinguishing between the two diseases for early diagnosis and targeted management, ultimately improving patient outcomes.

## 1. Introduction

Fever of Unknown Origin (FUO) is a common clinical challenge for physicians. There are many causes of FUO in children, including infections, rheumatologic diseases, auto-inflammatory diseases, and even neoplasms [1]. Many etiologies are universal, yet some are more geographically and population-dependent by nature. Brucellosis and rickettsiosis are zoonotic diseases endemic to overlapping regions, including the Middle East, parts of Asia, and the Mediterranean Basin [2,3,4]. Brucellosis, caused by *Brucella* species, accounts for over 500,000 cases globally each year. Rickettsiosis, caused by Rickettsia species, accounts for more than 1 million cases annually in endemic areas, according to the Centers for Disease Control and Prevention (CDC) [5]. Both diseases pose significant public health challenges in endemic regions and are particularly common among the Bedouin population in southern Israel [6]. *Brucella* is a small Gram-negative coccobacillus, causing a variable and unspecific clinical presentation, with *Brucella melitensis* being the most virulent species in humans [7]. The disease spreads from animals (mostly sheep and camels) to humans by direct exposure to infected animals and their fomites or the ingestion of their unpasteurized milk. The clinical presentation usually consists of fever accompanied by weakness, loss of appetite, and arthralgia [6,8,9]. The *Rickettsia* genus is an obligatory intracellular, Gram-negative bacterium. There are many species of *Rickettsia* worldwide, causing different zoonotic illnesses with variable clinical courses [10]. The two predominant species in Israel are *Rickettsia conorii* and *Rickettsia typhi* [11,12]. While *R. conorii* is responsible for Mediterranean spotted fever (MSF), *R. typhi* is the etiological agent of murine typhus (MT). Both are arthropod-borne diseases, transmitted by the brown dog tick, *Rhipicephalus sanguineus*, and by the rat flea *Xenopsylla cheopis*, respectively [10,12,13,14]. As the name suggests, MSF usually causes fever followed by rash, and though it is usually a self-limiting disease, it can lead to organ failure and even death [12,15]. The clinical course of MT is less specific, with common symptoms being fever, rash, headache, and gastrointestinal (GI) manifestations [10,13,14].

The comparison between these diseases lies in their zoonotic origin and their overlapping clinical presentations, predominantly fever accompanied by malaise. This resemblance is likely driven by shared pathogenic mechanisms, including immune-mediated responses to bacterial antigens. Furthermore, these infections often exhibit comparable laboratory features, such as leukopenia, thrombocytopenia, and elevated liver enzymes, reflecting systemic inflammation and organ involvement [6,12,13,14,15]. Additionally, both diseases share antimicrobial agents used in treatment protocols, primarily doxycycline. However, brucellosis generally requires a prolonged treatment regimen for effective management [16,17,18].

Although it might be easier for experienced clinicians to make the distinction between these illnesses, some cases may prove challenging to diagnose early. The similarities may cause confusion for many physicians, leading to misdiagnoses and wrong or partial empiric treatment. Serious complications of brucellosis, which are much rarer in children than in adults, include endocarditis and neurological involvement, such as meningoencephalitis, cranial nerve palsies, and peripheral neuropathy. These complications, occurring in less than 5% of cases, underscore the importance of accurate and early diagnosis to prevent long-term effects [8,19,20,21,22]. Although MT typically causes a less severe disease, organ-specific complications are possible and include pneumonitis, hepatitis, meningoencephalitis, renal failure, and, in rare cases, endocarditis [14,23]. On the other hand, MSF shows higher rates of mortality and life-threatening complications. These include cardiac symptoms, cerebral infarct, meningoencephalitis, renal failure, and even secondary hemophagocytic lymphohistiocytosis (HLH) [15]. Early diagnosis and prompt empirical treatment may assist in avoiding such complications.

We conducted a retrospective study comparing brucellosis and rickettsiosis in children, focusing on their demographic, clinical, and laboratory characteristics. This research addresses a notable gap for physicians in endemic regions worldwide, as there are limited data directly comparing these two diseases in children. By enhancing our understanding of their distinguishing features, we aim to provide valuable insights for improving the diagnosis and management of these infections in children, ultimately guiding clinicians toward more effective treatment strategies.

## 2. Methods

### 2.1. Study Design

A retrospective cohort study was conducted in the pediatric emergency department (PED) and the pediatric wards of the Soroka University Medical Center (SUMC) between 2005 and 2020. Data were collected from the SUMC computerized database. The study included all relevant subjects who tested positive for either rickettsia or brucella. The study was approved by the SUMC Helsinki committee, with approval number 0255-23-SOR-C.

### 2.2. Study Population 

The study included all children < 18 years of age with a positive test for either rickettsia or brucella between 2005 and 2020. All children participating in this research had appeared with a clinical presentation suggestive of either brucellosis or rickettsiosis (i.e., FUO), thus making the diagnostic process challenging, leading the treating physician to test for both diseases. 

### 2.3. Diagnosis of Brucellosis 

**Figure 1:**
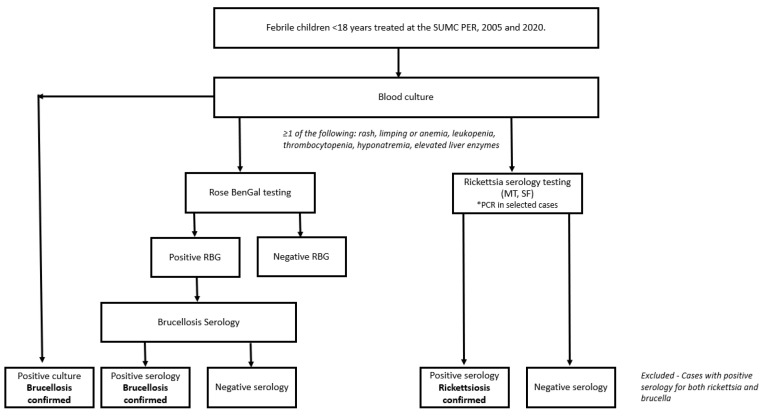
Flowchart summarizing the diagnostic and exclusion criteria.

The case definition for brucellosis included laboratory confirmation through blood culture or specific serologic testing. At our center, diagnosis began with initial screening with the brucella (Rose Bengal) test by Atlas Medical™ (Blankenfelde-Mahlow, Berlin, Germany), which detects Brucella antibodies in serum [24,25]. Positive cases were then confirmed with additional tests, such as the standard agglutination test (SAT) or the enzyme-linked immunosorbent assay (ELISA), both of which measure antibody titers against brucella species. An SAT titer of ≥1:160 or elevated IgG or IgM levels in ELISA are generally indicative of an active or recent brucella infection. These criteria, alongside clinical findings, form a reliable case definition for brucellosis (Figure 1).

### 2.4. Diagnosis of Rickettsiosis

In the current study, we utilized serologic testing to accurately diagnose rickettsial infections and distinguish between the two species that are common in Israel: *Rickettsia conorii* and *Rickettsia typhi*. In a small number of cases (<10% of all rickettsiosis cases), nested PCR was employed to detect *Rickettsia* DNA directly in blood samples, allowing for the rapid and specific identification of the pathogen during the acute phase of infection. The PCR testing targeted conserved regions of the *gltA* (citrate synthase) and *ompA* (outer membrane protein A) genes, using primers previously validated for their high specificity and sensitivity in identifying *Rickettsia* species [26]. PCR testing for rickettsiosis allows the achievement of high diagnostic value (sensitivity reaching to 80–100%), especially in the acute phase, when timely and accurate diagnosis can guide early empirical treatment [27]. Serologic testing, primarily via indirect immunofluorescence assay (IFA), was conducted in all included patients to measure antibody titers against rickettsia antigens. Notably, there is the potential for cross-reactivity in serologic tests, as previously reported, and, thus, we did not separate rickettsiosis cases into two distinct groups (i.e., MT and MSF) but, rather, grouped them for analysis [28,29,30]. 

We excluded cases where a positive result was received for both rickettsia and brucella.

### 2.5. Variables and Data Collection 

Data from the SUMC computerized database included various demographic, clinical, laboratory, and outcome-related variables. Demographic information included age, gender, ethnicity, and seasonality. For seasonality definition, tests conducted between September and February were classified as the “cold season”, while those conducted from March to August were categorized as the “warm season”.

Disease history data included reports of symptoms such as fever, rash, limp, headache, gastrointestinal complaints (e.g., abdominal pain, vomiting, and diarrhea), respiratory symptoms, seizures, and loss of consciousness. Physical examination findings included body temperature, a rash, and joint swelling or tenderness. Neurological examination findings included an altered mental status and nuchal rigidity. Laboratory data included hemoglobin levels, blood leukocyte count, platelet count, liver transaminases (AST and ALT), CRP levels, sodium levels, kidney function tests (e.g., creatinine and urea), and blood cultures where applicable. Outcome variables included the hospitalization duration, the number and the duration of antibiotics administered, and mortality rates. 

### 2.6. Quantitative Variables and Statistical Methods

Descriptive and univariate statistical analyses were performed using SPSS version 29. Descriptive statistics summarized the characteristics of positive rickettsia and brucella cases, using means and standard deviations for normally distributed continuous variables and medians with interquartile ranges for non-normally distributed variables. Univariate analyses were conducted to assess differences between positive rickettsia and brucella cases. For categorical variables, a two-tailed Chi-squared test was used to compare proportions as it is appropriate when the sample size is large enough to ensure expected cell counts above 5 [31]. For continuous variables, Student’s *t*-test was applied when the data were normally distributed, as this test compares the means of two independent groups under the assumption of normality. If the data were not normally distributed, then the Mann–Whitney U test was employed as a non-parametric alternative. This test is particularly useful for ordinal data or continuous data that are skewed [32]. Statistical significance was set at *p* < 0.05. 

A logistic regression model was employed to assess potential factors, covariates, and confounders associated with the diagnosis of brucellosis and rickettsiosis. Two separate multivariate logistic regression models were constructed, incorporating variables such as demographic factors, clinical symptoms (e.g., rash, limping), and laboratory findings (e.g., white blood cell count, platelet count, and CRP levels).

## 3. Results

### 3.1. Demographic Parameters

**Figure 2:**
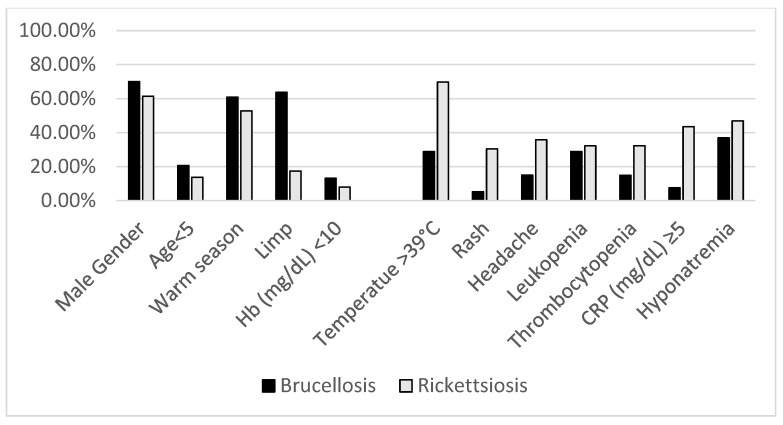
Comparison of demographic and clinical characteristics of brucellosis and rickettsiosis.

Overall, 440 patients with brucellosis and 335 patients with rickettsiosis (281 were diagnosed with MT and 54 with MSF) were included. Less than 1% of cases (n = 4) were excluded due to a co-morbidity diagnosis of rickettsiosis and brucellosis (Table 1, Figure 2).

The majority of patients in both groups were of Bedouin ethnicity, with higher proportions among brucellosis patients (99.5% vs. 90.7%, *p* < 0.001). Gender distribution differed between the groups, with higher proportions of females in the rickettsiosis group (38.6% vs. 30%, *p* = 0.01). Brucellosis patients were significantly younger, with a higher proportion of children <5 years of age (20.6% vs. 13.7%, *p* = 0.01). 

### 3.2. Seasonality

A seasonal pattern was observed in both groups, with more cases occurring during the warm season (March to August). The brucellosis group had a higher proportion of cases during this period compared with the rickettsiosis group (60.9% vs. 52.8%, *p* = 0.03).

### 3.3. Clinical Parameters

Rickettsiosis patients presented with a higher body temperature upon arrival to the PED, with a mean temperature of 39.4 ± 0.96 °C, compared with 38.27 ± 1.16 °C in the brucellosis group (*p* < 0.001). Higher-grade fever (≥39 °C) was associated with rickettsiosis (69.8% vs. 28.8%, *p* < 0.001). 

Rash was associated with rickettsiosis (30.4% vs. 5.2%, *p* < 0.001), reflecting the fact that rickettsial infection targets endothelial cells. Similarly, headache was more frequent in rickettsiosis (35.8% vs. 15.0%, *p* < 0.001), likely due to the systemic inflammatory response, which can cause vascular inflammation (vasculitis) and increased intracranial pressure. In contrast, limping was more routinely observed in the brucellosis group (63.7% vs. 17.4%, *p* < 0.001). Joint involvement in brucellosis, often due to inflammation or arthritis, can cause pain and reduced mobility, which may manifest as limping in affected children.

Diarrhea (11.6% vs. 5.7%, *p* = 0.004), abdominal pain (21.8% vs. 7.5%, *p* < 0.001), and vomiting (38.5% vs. 12.3%, *p* < 0.001) were significantly more prevalent in rickettsiosis compared with brucellosis, reflecting greater gastrointestinal involvement (Supplementary Table S1). 

Neurological signs and symptoms, including seizures or loss of consciousness (6.3% vs. 1.8%, *p* = 0.002) and nuchal rigidity (3.0% vs. 0.2%, *p* = 0.001), were more commonly observed in rickettsiosis, suggesting possible central nervous system involvement. Additionally, rickettsiosis was associated with both upper respiratory tract infections (25.4% vs. 13.0%, *p* < 0.001) and lower respiratory tract infections (5.4% vs. 2.5%, *p* = 0.05).

### 3.4. Laboratory Features

Hemoglobin (Hb) levels were lower among brucellosis patients, with a mean Hb of 11.44 ± 1.47 mg/dL compared with 12.25 ± 1.63 mg/dL in the rickettsiosis group (*p* < 0.001). Hemoglobin levels <10 mg/dL was associated with brucellosis (13.1% vs. 7.9%, *p* = 0.025).

Thrombocytopenia (platelet count <150,000/mm^3^) was more prevalent in the rickettsiosis group than in the brucellosis group (32.3% vs. 14.8%, *p* < 0.001). Similarly, C-reactive protein (CRP) levels were significantly higher in the rickettsiosis group, with a mean of 6.49 ± 7.68 mg/dL, compared with 1.74 ± 2.12 mg/dL in the brucellosis group (*p* < 0.001). Additionally, hyponatremia (sodium levels < 135 mEq/L) was associated with rickettsiosis (46.9% vs. 36.9%, *p* = 0.008).

### 3.5. Outcomes

The mean duration of hospitalization was significantly shorter in the rickettsiosis group, with an average stay of 4.18 ± 2.58 days, compared with 6.15 ± 3.83 days in the brucellosis group (*p* < 0.001). The number of antimicrobial agents used per patient was lower in the rickettsiosis group compared with the brucellosis group (0.53 ± 0.86 vs. 1.89 ± 0.85, *p* < 0.001). 

Doxycycline was prescribed to a significantly higher proportion of brucellosis patients compared with rickettsiosis patients (63.1% vs. 48.9%, *p* < 0.001). Gentamicin was used in 92.5% of brucellosis patients, compared with 5.1% of the rickettsiosis patients (*p* < 0.001). Similarly, co-trimoxazole was used in 29.5% of brucellosis patients, while none of the rickettsiosis patients received this antibiotic (*p* < 0.001).

The overall duration of antimicrobial treatment was longer in the brucellosis group, with a mean of 38.52 ± 11.7 days, compared with 3.56 ± 3.98 days in the rickettsiosis group (*p* < 0.001).

Two cases of mortality were reported in brucellosis patients, while no cases of mortality were reported in rickettsiosis patients.

### 3.6. Multivariate Analysis

Multivariate analysis identified several factors significantly associated with the diagnosis of brucellosis and rickettsiosis in febrile children (see Table 2). For brucellosis, key factors included limping (OR 7.27, 95% CI 5.15–10.38, *p* < 0.001), hemoglobin levels <10 mg/dL (OR 2.01, 95% CI 1.14–3.64, *p* = 0.018), age <5 years (OR 1.95, 95% CI 1.25–3.07, *p* = 0.003), occurrence during the warm season (March–August) (OR 1.84, 95% CI 1.31–2.59, *p* < 0.001), and male gender (OR 1.57, 95% CI 1.10–2.25, *p* = 0.012).

In contrast, rickettsiosis was significantly associated with the presence of a rash (OR 9.06, 95% CI 3.91–24.9, *p* < 0.001), elevated CRP levels ≥5 mg/dL (OR 4.03, 95% CI 1.86–9.81, *p* < 0.001), headache (OR 3.01, 95% CI 1.75–5.30, *p* < 0.001), thrombocytopenia with platelet counts <150,000/mm^3^ (OR 2.61, 95% CI 1.23–6.06, *p* = 0.017), white blood cell counts <5000/mm^3^ (OR 1.88, 95% CI 1.19–2.98, *p* = 0.006), and a high fever (temperature ≥39.0 °C) (OR 1.66, 95% CI 1.03–2.68, *p* < 0.001). Hyponatremia showed no significant association with rickettsiosis (OR 0.79, 95% CI 0.48–1.28, *p* = 0.33).

## 4. Discussion

Brucellosis and rickettsiosis often present with similar symptoms, mainly FUO, posing diagnostic and management challenges when febrile children present to the PED in endemic areas. In this retrospective cohort study, we identified several differences between pediatric patients with brucellosis and rickettsiosis. *Brucella* patients were almost exclusively Bedouins, usually younger, and more likely to present with limping, while rickettsiosis was associated with higher-grade fever, rash, and headache, as well as with gastrointestinal, respiratory, and neurologic signs and symptoms. Anemia was prevalent in brucellosis, whereas thrombocytopenia and hyponatremia were seen more often in rickettsiosis. Rickettsiosis patients also had shorter mean hospital stays and required fewer antibiotics. These distinctions may offer valuable guidance for early clinical management in the PED setting.

Our study found significant demographic differences between brucellosis and rickettsiosis patients. Brucellosis was almost exclusive to Bedouin children (99.5%), while rickettsiosis included a small percentage of Jewish patients (9.3%). This may reflect different environmental exposures or lifestyle factors, as Bedouin individuals are more exposed to unpasteurized milk and domestic animals [33,34,35]. This issue should raise clinical suspicion and holds public health significance in efforts to reduce infections among children in an agrarian society, for example, through education. Additionally, male patients were more predominant in the brucellosis group compared with the rickettsiosis group, aligning with previous reports indicating a higher prevalence of brucellosis in male individuals due to occupational exposures, such as contact with livestock [36]. Moreover, febrile children of a younger age, particularly those from populations with known risk factors for brucellosis, should raise clinical suspicion for early diagnosis.

Rash was significantly more common in rickettsiosis. This finding suggests that the presence of a rash may serve as a clinical marker to differentiate between these infections. Specifically, most of our rickettsiosis cases were caused by *Rickettsia typhi* (causing murine typhus) which is known to present with a lower rate of rash compared with *Rickettsia conorii*. This aligns with findings from previous work from southern Israel, which discusses the clinical nuances of *R. typhi* and its associated rash patterns, highlighting the importance of considering rash as a diagnostic feature in distinguishing between these infections [37].

Brucellosis patients were more likely to present with a limp (63.7%) compared with rickettsiosis patients (17.4%), likely reflecting the fact that joint involvement is often seen in brucellosis. These findings synchronize with previous studies that describe brucellosis as commonly presenting with musculoskeletal symptoms, including limping and joint pain [38,39], while rickettsiosis is frequently associated with rash and other systemic manifestations [40,41]. 

The higher mean temperature observed in the rickettsiosis group suggests that rickettsiosis patients present to the PED early in the course of the disease, at the acute phase of the disease, while brucellosis often presents in the PED with sub-acute or even chronic disease. Headache was more commonly observed in rickettsiosis patients. We hypothesize that this may reflect higher rates of aseptic meningitis in this group. Notably, few data are available regarding rates of meningitis in both diseases, since lumbar puncture is performed in only a small fraction of the patients. As for laboratory findings, the two diseases showed distinct profiles. Anemia, as found previously, was more common in brucellosis [42]. Leukopenia was detected in both groups, at similar rates. However, aligned with current knowledge, thrombocytopenia and hyponatremia were significantly more common in rickettsiosis patients [43,44]. The increased rates of thrombocytopenia and hyponatremia in rickettsiosis may be attributed to its more systemic nature, often involving multiple organs, including the kidneys, which play a key role in maintaining sodium balance [45,46]. Hyponatremia was identified in 36.9% of brucellosis patients, a phenomenon that some of the literature suggests may result from electrolyte imbalances associated with the disease [47,48]. C-reactive protein (CRP) levels, a marker commonly associated with rickettsial infections, consistent with findings from previous studies, were significantly higher in the rickettsiosis group [49]. The elevated CRP levels in rickettsiosis likely reflect its acute nature, prompting rapid testing due to fast onset and inflammation. In contrast, brucellosis typically follows a subacute course, explaining the lower CRP levels seen [41,50]. Treatment outcomes significantly differed between the two groups. Rickettsiosis patients had shorter hospital stays compared with brucellosis patients. As expected, rickettsiosis patients required fewer antimicrobial agents than brucellosis patients, indicating the need for more aggressive combination treatment in brucellosis [18,51]. While doxycycline was commonly used for both infections, it was administered more frequently in brucellosis cases (63.1% vs. 48.9%). The longer duration of antimicrobial treatment for brucellosis reflects the chronic nature of brucellosis, which often requires extended therapy to prevent relapse [16,51].

Our findings emphasize the need for prompt empirical treatment in children with suspected rickettsiosis or brucellosis in endemic areas. Clinical predictors should guide therapy to prevent delays. Early treatment decisions, based on demographic, clinical, and laboratory factors, can be made even before serological confirmation. For instance, a rash, hyponatremia, thrombocytopenia, and elevated CRP suggest rickettsiosis, warranting doxycycline treatment. Brucellosis should be suspected in febrile children with a limp, anemia, and younger age, with treatment deferred until diagnosis confirmation. We recommend developing diagnostic flowcharts and treatment protocols to integrate these findings, with an emphasis on urgent doxycycline use for rickettsiosis cases.

This study has several limitations. As a single-center, retrospective study, it relies on the accuracy and completeness of medical records, which may introduce bias. Changes in diagnostic methods over time could have influenced the results. The increasing use of PCR for rickettsiosis diagnosis may have reduced the false negatives seen with serological tests, which have limitations in detecting early infections or distinguishing cross-reactivity [28,30]. However, the study strengths include a large cohort of 775 children over 15 years and a thorough review of the computerized database to capture all clinical data.

## 5. Conclusions

In conclusion, this study highlights the distinct demographic, clinical, and laboratory profiles of brucellosis and rickettsiosis. These differences are important in guiding early clinical decision-making and selecting appropriate empirical treatment in emergency department settings in endemic regions. By establishing diagnostic strategies focusing on clinical characteristics, physicians can reduce diagnostic delays and guide empirical treatment. Rickettsiosis patients, who often present with high-grade fever, rash, headache, hyponatremia, elevated CRP levels, and thrombocytopenia, may benefit from the prompt initiation of doxycycline. In contrast, it may be appropriate to ascertain the diagnosis in brucellosis patients, typically exhibiting a limp and anemia, before initiating a prolonged combination therapy. 

## Figures and Tables

**Table 1:** Comparison of brucellosis and rickettsiosis.

		Brucellosis N = 440	Rickettsiosis N = 335	*p*-Value
Demographics	Jewish (N, %)	2 (0.5%)	31 (9.3%)	<0.001
	Bedouin (N, %)	438 (99.5%)	304 (90.7%)	<0.001
	Gender—male (N, %)	308 (70.0%)	206 (61.4%)	0.01
	Gender—female (N, %)	132 (30.0%)	129 (38.6%)	0.01
	Age (years); mean ± SD	9.9 ± 4.9	10.1 ± 5.0	<0.001
	Age (years) < 5; (N, %)	91 (20.6%)	46 (13.7%)	0.01
Seasonality	Warm season (March–August) (N, %)	268 (60.9%)	177 (52.8%)	0.03
	Cold season (September–February) (N, %)	172 (39.1%)	156 (47.2%)	0.03
Clinical parameters	Temperature at ER; mean ± SD	38.3 ± 1.16	39.4 ± 0.96	<0.001
	Temperature ≥39.0; (N, %)	127 (28.8%)	234 (69.8%)	<0.001
	Rash; (N, %)	23/440 (5.2%)	102 (30.4%)	<0.001
	Limp; (N, %)	250/392 (63.7%)	58/334 (17.4%)	<0.001
	Headache; (N, %)	66/440 (15.0%)	120/335 (35.8%)	<0.001
Laboratory features	Hb (mg/dL); mean ± SD	11.44 ± 1.47	12.25 ± 1.63	<0.001
	Hb (mg/dL) < 10; (N, %)	57/434 (13.1%)	26/331 (7.9%)	0.025
	WBC (×1000/mm^3^); mean ± SD	8.29 ± 28.6	7.42 ± 4.44	0.58
	WBC (×1000/mm^3^) < 5; (N, %)	127/440 (28.9%)	108/335 (32.2%)	0.34
	Platelets (×1000/mm^3^); mean ± SD	254.9 ± 109.9	210.4 ± 107.9	<0.001
	Platelets (×1000/mm^3^) < 150; (N, %)	64/433 (14.8%)	108/334 (32.3%)	<0.001
	ALT (U/L); mean ± SD	41.35 ± 46.18	45.51 ± 155.44	0.63
	ALT (U/L) ≥ 45; (N, %)	89/369 (24.1%)	76/309 (24.6%)	0.93
	AST (U/L); mean ± SD	56.66 ± 42.29	70.20 ± 282.71	0.37
	AST (U/L) ≥ 45; (N, %)	201/367 (54.8%)	129/297 (43.4%)	0.004
	CRP (mg/dL); mean ± SD	1.74 ± 2.12	6.49 ± 7.68	<0.001
	CRP (mg/dL) ≥ 5; (N, %)	12/159 (7.5%)	124/285 (43.5%)	<0.001
	Sodium (mEq/L); mean ± SD	135.43 ± 2.90	134.48 ± 3.37	<0.001
	Sodium (mEq/L) < 135; (N, %)	147/398 (36.9%)	153/326 (46.9%)	0.008
Clinical outcomes	Hospitalization duration (days); Mean ± SD	6.15 ± 3.83	4.18 ± 2.58	<0.001
	Number of antimicrobial agents used; mean ± SD	1.89 ± 0.85	0.53 ± 0.86	<0.001
	Doxycycline used; (N, %)	278 (63.1%)	164 (48.9%)	<0.001
	Gentamicin used; (N, %)	407 (92.5%)	17 (5.1%)	<0.001
	Co-trimoxazole used; (N, %)	130 (29.5%)	0 (0%)	<0.001
	Antimicrobial treatment duration (days); mean ± SD	38.52 ± 11.7	3.56 ± 3.98	<0.001
	Mortality; mean ± SD	2 (0.5%)	0 (0%)	0.50

**Table 2:** Multivariate analysis of factors associated with diagnosis of brucellosis and rickettsiosis in febrile children.

	Factors	Odds Ratio	95% Confidence Intervals	*p*-Value
Factors Associated with Diagnosis of Brucellosis	Limp	7.27	5.15–10.38	<0.001
	Hb (mg/dL) < 10	2.01	1.14–3.64	0.018
	Age (years) < 5	1.95	1.25–3.07	0.003
	Warm season (March–August)	1.84	1.31–2.59	<0.001
	Gender—male	1.57	1.10–2.25	0.012
Factors Associated with Diagnosis of Rickettsiosis	Rash	9.06	3.91–24.9	<0.001
	CRP (mg/dL) ≥ 5	4.03	1.86–9.81	<0.001
	Headache	3.01	1.75–5.30	<0.001
	Platelets (×1000/mm^3^) < 150	2.61	1.23–6.06	0.017
	WBC (×1000/mm^3^) < 5	1.88	1.19–2.98	0.006
	Temperature ≥ 39.0	1.66	1.03–2.68	<0.001
	Sodium (mEq/L) < 135	0.79	0.48–1.28	0.33

## Data Availability

The data presented in this study are available on request from the corresponding author due to privacy and ethical restrictions, as the dataset contains sensitive patient information that cannot be shared publicly.

## Supplementary Materials

The following supporting information can be downloaded at: https://www.mdpi.com/article/10.3390/jcm14051465/s1, Table S1: Comparison of additional signs and symptoms of brucellosis and rickettsiosis.
