# Supplementary material for: Comparison of Brucellosis and Rickettsiosis in Children: A Retrospective Cohort Study
Source: J Clin Med. 2025 Feb 21;14(5):1465. doi: 10.3390/jcm14051465 (PMC11900969; doi:10.3390/jcm14051465)
Supplement: Supplementary file 1 [file jcm-14-01465-s001.zip › jcm-3418133-supplementary.pptx]

## Slide 1
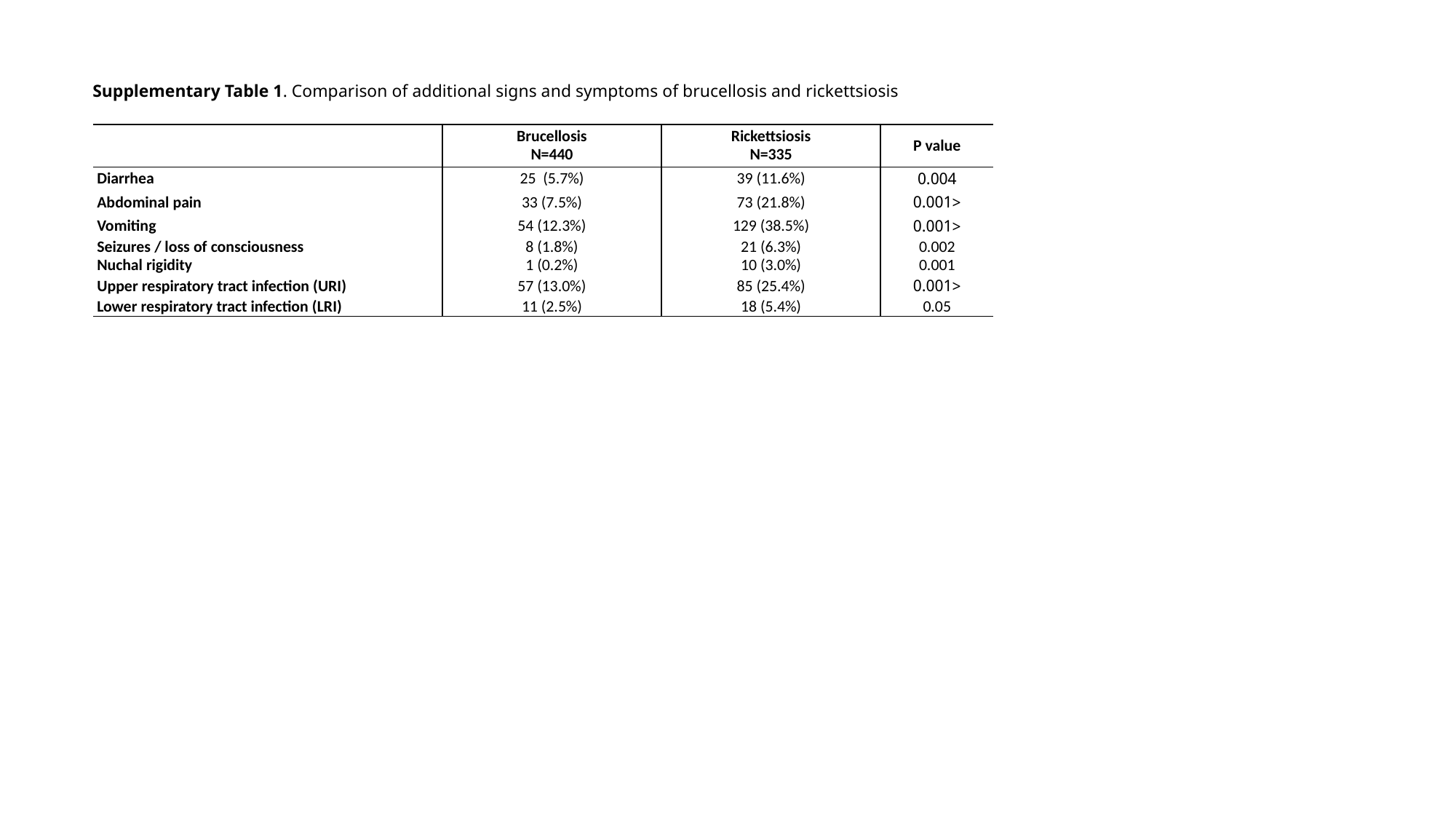

Supplementary Table 1. Comparison of additional signs and symptoms of brucellosis and rickettsiosis
| | Brucellosis N=440 | Rickettsiosis N=335 | P value |
| --- | --- | --- | --- |
| Diarrhea | 25 (5.7%) | 39 (11.6%) | 0.004 |
| Abdominal pain | 33 (7.5%) | 73 (21.8%) | <0.001 |
| Vomiting | 54 (12.3%) | 129 (38.5%) | <0.001 |
| Seizures / loss of consciousness | 8 (1.8%) | 21 (6.3%) | 0.002 |
| Nuchal rigidity | 1 (0.2%) | 10 (3.0%) | 0.001 |
| Upper respiratory tract infection (URI) | 57 (13.0%) | 85 (25.4%) | <0.001 |
| Lower respiratory tract infection (LRI) | 11 (2.5%) | 18 (5.4%) | 0.05 |
